# Epidemic Rumination and Resilience on College Students' Depressive Symptoms During the COVID-19 Pandemic: The Mediating Role of Fatigue

**DOI:** 10.3389/fpubh.2020.560983

**Published:** 2020-12-09

**Authors:** Baojuan Ye, Xiuxiu Zhou, Hohjin Im, Mingfan Liu, Xin Qiang Wang, Qiang Yang

**Affiliations:** ^1^Center of Mental Health Education and Research, School of Psychology, Jiangxi Normal University, Nanchang, China; ^2^Department of Psychological Science, University of California, Irvine, Irvine, CA, United States; ^3^School of Education, Jiangxi Normal University, Nanchang, China

**Keywords:** COVID-19, epidemic rumination, depression, fatigue, resilience

## Abstract

The restriction of numerous sectors of society and the uncertainty surrounding the development of the COVID-19 pandemic have resulted in adverse psychological states to college students isolated at home. In this study, we explored the mediating role of fatigue in the effects of epidemic rumination and resilience on depressive symptoms as well as how epidemic rumination and resilience may interact with one another. A large sample of Chinese college students (*N* = 1,293) completed measures on epidemic rumination, resilience, fatigue, and depressive symptoms. Results indicated depressive symptomology was positively predicted by epidemic rumination while negatively predicted by resilience. In both cases, fatigue partially mediated these effects and positively predicted depressive symptoms. Unexpectedly, epidemic rumination and resilience interacted in a manner where the effect of rumination on fatigue became stronger as resiliency increased. Theoretical and practical implications are provided to further interpret the results.

## Introduction

The heavy losses to the lives and property of people around the world from the global outbreak and spread of COVID-19 has induced severe psychological trauma to those affected. China was one of the earliest countries to be affected by COVID-19 and likewise one of the first to implement widescale measures to curb viral spread. In an effort to limit the spread among youth on college campuses, the Ministry of Education in China extended Winter recess and postponed the start of Spring semester. For Chinese college students, prolonged time at home with limited ability to go outside meant doing one's part to stop the spread of COVID-19. However, this came at the cost of abating their participation in normative social activities, such as meeting friends or participating in extracurricular activities. Such public health measures have led to a downstream torrent of negative mental health outcomes. Indeed, several studies have found that COVID-19 related stressors accrued a myriad of negative effects on mental health, such as inducing symptoms of both anxiety and depression [e.g., (1–3)].

Although few in number, these early studies have troubling implications for the general public knowing that depression has been linked to high rates of morbidity, recurrence, disability, and suicide (4), and has since become one of the major factors endangering human health (5). While some evidence suggests that the physiological damage caused by depression may be short-lived, the psychological effects may be long-term (5). As is the case with many large-scale disasters, the negative mental effects COVID-19 induced on its general populace is rudimentary. Despite this, little attention has been given to the mental health status of younger individuals within the COVID-19 body of research (6). Because college students are often at the developmental stage in which they transition from adolescence to adulthood, this population may be particularly at risk. As the world continues its fight against the pandemic, it remains highly imperative to probe the antecedents of the onset of depressive symptoms amongst college students to design effective social interventions (7–9). Contributing to this significant gap in literature, we explored how resiliency and COVID-19 specific rumination may, respectively, mitigate and exacerbate fatigue, which in turn, increases the severity of depressive symptoms. Further, we examined whether resiliency and rumination interacted in a manner such that resiliency buffered the effect of rumination on fatigue.

### Epidemic Rumination and Depression

One's susceptibility to depression is partly contingent upon individual factors that can play a promotive role in the occurrence and development of the mental illness (10). Specifically, ruminative response style is argued to be a key risk factor for depression (11). Rumination is characterized by persistent and passive cognitive deliberation of negative stressors and events, ultimately aggravating preexisting depressive symptoms (12, 13) and crippling one's abilities for positive problem-solving (14–16). Those who exhibit greater rumination have been documented to experience more intense negative emotions (4, 17–19) and sense of hopelessness (20). Accordingly, rumination is largely in part considered a maladaptive response to stressors, given its large consumption of cognitive resources.

As rumination hinders adaptive problem-solving (21) and induces greater hopelessness (20), individuals may further lose the motivation to tackle the source of the issue, resulting in prolonged depressive symptoms (22). Early evidence of the role of rumination on stress consequences amid the COVID-19 pandemic has generally supported prior findings [e.g., (3, 23)]. However, these studies have measured general ruminative tendencies within the individual. Because it may be possible that individuals that otherwise do not engage in rumination during normative times developed ruminative tendencies specific to only COVID-19, we contend that a more target-specific approach may be necessary to better capture the cognitive responses to the novel virus. Following the definition of general rumination (24), we define *epidemic rumination* as ruminative tendencies specifically pertaining to the events surrounding COVID-19. Given evidence of the link between rumination and depression (15, 25), individuals with high levels of epidemic rumination may exhibit greater depressive symptomology (4, 17–19).

### Resilience and Depression

While epidemic rumination is a risk factor for depression, there are also those who show resiliency to life stressors. Resiliency refers to one's ability to actively adapt and cope with the impact of stress or trauma (26), showing adversity in the face of setbacks (27, 28) and generally adept at maintaining or promoting positive mental health outcomes (29). Accordingly, those with greater resiliency generally tend to exhibit lower levels of depression (30–32). In the midst of the COVID-19 pandemic, a slew of recent studies has shown that resilient healthcare professionals experienced lower anxiety, posttraumatic stress, and depression (33, 34). Among the general populace, similar patterns emerged (30, 32, 35, 36). In other words, those who were able to adaptively cope with COVID-19 related stressors were better equipped to attenuate the onset of emotional distress consequences (37, 38). Thus, individuals able to remain steadfast and optimistic in spite of the current turbulent state of the world may be less likely to suffer from depressive symptoms.

### The Mediating Role of Fatigue

With the prolonged nature and intensity of COVID-19, however, many individuals will naturally experience some form of fatigue, whether that be physical or psychological (39). This may be particularly the case for college students who are often not adept at handling sudden and large life stressors (40, 41), putting them at greater risk for developing depressive symptoms. As fatigue is a common byproduct of depleted psychological resources, risk factors [e.g., rumination; (42, 43)] and protective factors [e.g., resiliency; (44)] for said resources may, respectively, exacerbate and mitigate the onset of physical and psychological fatigue among individuals. Specifically, Luceño-Moreno et al. (34) found a strong negative relation between resilience and experience of emotional exhaustion among those impacted by COVID-19. In this regard, resilience may serve to not only directly reduce the experience of fatigue, but also serve to buffer the negative consequences of observed risk factors (e.g., epidemic rumination) (26, 45) through cognitive reappraisal (46). This may be especially critical given the rudimentary nature of fatigue in its risk to the negative consequences of life stressors (47), such as depression (48–50).

### The Present Study

**Figure 1:**
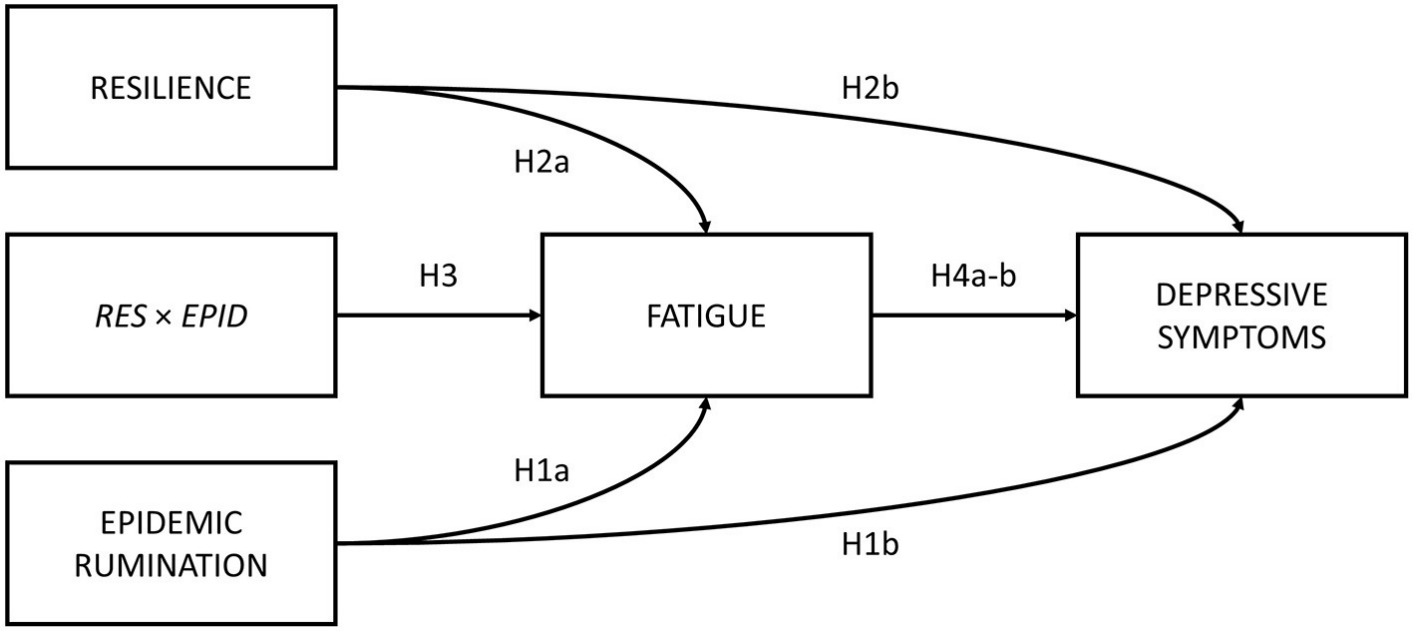
Proposed conceptual mediated model.

The present study sought to first examine the roles of epidemic rumination and resilience on depressive symptoms. Secondly, the current study examined the mediating role of fatigue in the aforementioned relations. Lastly, we examined whether epidemic rumination and resilience interacted with one another in their effect on fatigue. We proposed a conceptual model (Figure 1) and the following hypotheses:

**Hypothesis 1**. Epidemic rumination is positively related to (a) fatigue and (b) depressive symptoms.
**Hypothesis 2**. Resilience is negatively related to (a) fatigue and (b) depressive symptoms.
**Hypothesis 3**. Epidemic rumination and resilience significantly interact such that resilience buffers the effects of rumination on fatigue.
**Hypothesis 4**. Fatigue is positively related to (a) depressive symptoms and mediates the effect of (b) epidemic rumination and (c) resilience on depressive symptoms.

## Methods

### Participants

A large sample of 1,293 college students in China (*M*_age_ = 20.79, *SD*_age_ = 1.67, 52% Female) were recruited for this study. A total of 464 (35.90%) participants were first years, 271 (21.00%) were second years, 310 (24.00%) were third years, and 248 (19.20%) were fourth year students.

### Procedures

Participants were invited to participate in an anonymous, online survey on how COVID-19 has impacted their psychological state and behaviors. As Winter recess was in session during the data collection period of this study due to delayed start of the Spring semester, participants were surveyed through an online survey platform (“SurveyStar,” Changsha Ranxing Science and Technology, Shanghai, China). After giving informed consent, participants were directed to the psychological measurements.

### Measures

#### Epidemic Rumination

Epidemic rumination was measured via a 10-item COVID-19 abridged version of the Ruminative Response Scale [RRS; (51, 52)]. Prior studies using the RRS in Chinese samples have shown good reliability and validity [e.g., (53, 54)]. The current scale was comprised of two dimensions: (1) reflective pondering (e.g., “I often think about why COVID-19 turned out the way it did”) and (2) brooding (e.g., “I often go someplace alone to think about my feelings”). Each item was scored from 1 (*not at all true*) to 5 (*definitely true*), α = 0.76. Higher mean scores indicated greater levels of epidemic rumination. Confirmatory factor analysis (CFA) indicated acceptable fit, CFI = 0.92, TLI = 0.90, RMSEA = 0.08, SRMR = 0.05. See Appendix for all items.

#### Resilience

Resilience was measured via the Chinese version of the 10-item Connor-Davidson Resilience Scale (55), originally developed by Campbell-Sills and Stein (56). Prior use of this scale among Chinese participants showed good reliability and validity [e.g., (57)]. The scale was composed of ten items (e.g., “Able to adapt to change”), α = 0.94. All items were scored from 0 (*never*) to 4 (*always*). Higher mean scores indicated higher levels of resilience.

#### Fatigue

Fatigue was measured via the Chinese version of the Fatigue Assessment Scale (58), originally developed by Michielsen et al. (59). This scale has previously been used with Chinese participants with good reliability and validity [e.g., (58)]. The scale was composed of twenty items (e.g., “I have problems thinking clearly”) and each item was scored from 1 (*never*) to 5 (*always*), α = 0.86. Higher mean scores indicated greater levels of fatigue.

#### Depressive Symptoms

Depressive symptomology was measured via the Chinese version of the Center for Epidemiological Studies Depression Scale (60), originally developed by Radloff (61). Prior use of this scale among Chinese participants [e.g., (58, 62, 63)] have shown good reliability and validity. The scale was composed of twenty items and includes four dimensions: (1) depressed affect (e.g., “I felt lonely”), (2) positive affect (e.g., “I felt hopeful about the future”), (3) psychosomatic retardation (e.g., “I could not get ‘going”'), and (4) interpersonal relationships (e.g., “People were unfriendly”), α = 0.95. Each item was scored from 1 (*not at all true*) to 5 (*definitely true*). Higher mean scores indicated greater levels of depressive symptoms.

## Result

### Descriptive Statistics

Means, standard deviations, and Pearson correlations are given in Table 1. As expected, epidemic rumination was positively related to fatigue and depressive symptoms, and negatively related to resilience. Resilience was strongly negatively related to both fatigue and depressive symptoms. Fatigue was strongly positively related to depressive symptoms.

**Table 1:** Means, standard deviations, and correlations of the main study variables.

	***M***	***SD***	**1**	**2**	**3**	**4**
1. Epidemic rumination	2.97	0.54	–			
2. Fatigue	2.59	0.50	0.13^***^	–		
3. Depression	1.68	0.63	0.28^***^	0.65^***^	–	
4. Resilience	3.63	0.61	−0.14^***^	−0.40^***^	−0.74^***^	-

N = 1,293;

*** *p < 0.001*.

### Epidemic Rumination and Resilience on Depressive Symptoms: The Mediating Effect of Fatigue

**Figure 2:**
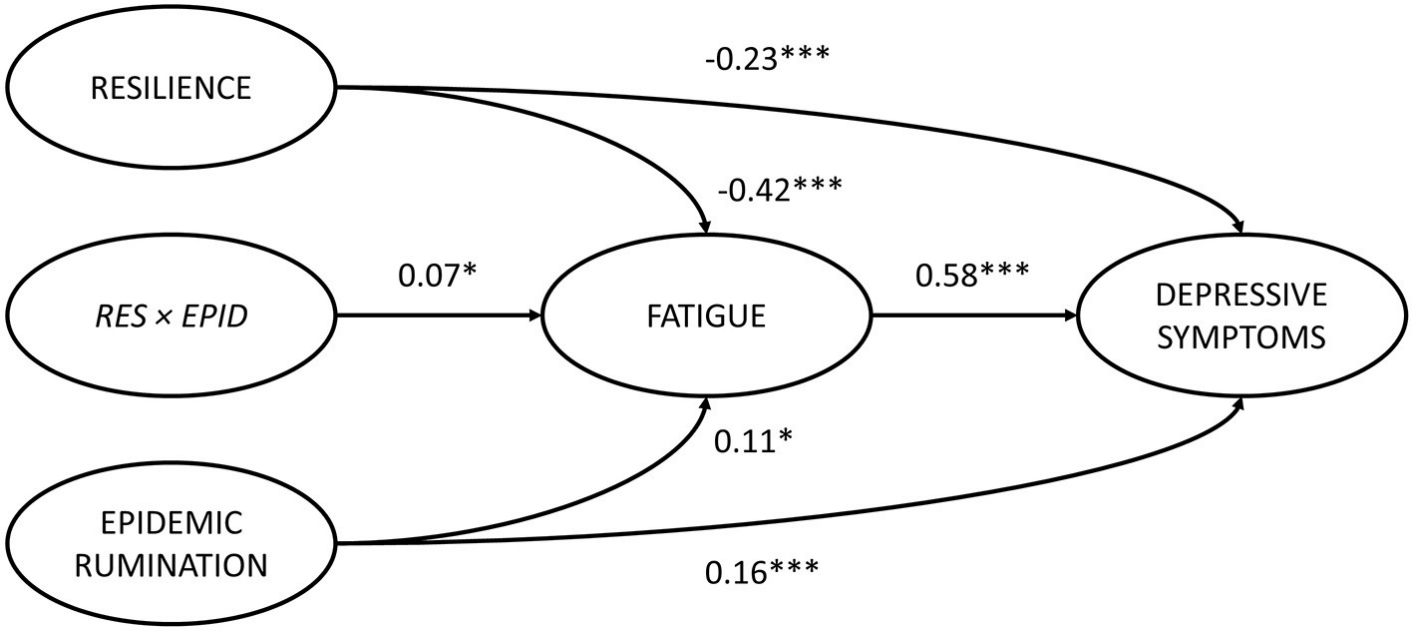
Path model of the proposed conceptual model. ***p < 0.001, **p < 0.01, *p < 0.05.

Structural equation modeling (SEM) through Mplus 8.3 (64) was used to analyze the mediating role of fatigue in the effects of epidemic rumination and resilience on depressive symptoms as well as the interaction between epidemic rumination and resilience on fatigue (Figure 2). The proposed model showed great fit (RMSEA = 0.05, SRMR = 0.04, CFI = 0.98, TLI = 0.98) based on field threshold standards (65, 66). Epidemic rumination was positively related to fatigue [γ = 0.11, *t* = 2.39, *p* = 0.017, 95% CI = (0.012, 0.176)] while resilience was negatively related to fatigue [γ = −0.42, *t* = −11.52, *p* < 0.001, 95% CI = (−0.426, −0.290)], supporting Hypotheses 1a and 2a. Moreover, epidemic rumination and resilience positively interacted in their relation to fatigue [γ = 0.07, *t* = 2.19, *p* = 0.029, 95% CI = (0.016, 0.154)]. Fatigue was a strong positive correlate of depressive symptoms [γ = 0.58, *t* = 19.12, *p* < 0.001, 95% CI = (0.619, 0.801)], supporting Hypotheses 4a-b that fatigue mediates the effect of epidemic rumination and resilience on depressive symptoms. Results also showed that even after controlling for fatigue, depressive symptomology was directly predicted by epidemic rumination [γ = 0.16, *t* = 5.15, *p* < 0.001, 95% CI = (0.110, 0.242)] and resilience [γ = −0.23, *t* = −10.073, *p* < 0.001, 95% CI = (−0.426, −0.290)], supporting Hypotheses 1b and 2b and suggesting that the mediation effect of fatigue was only partial.

**Figure 3:**
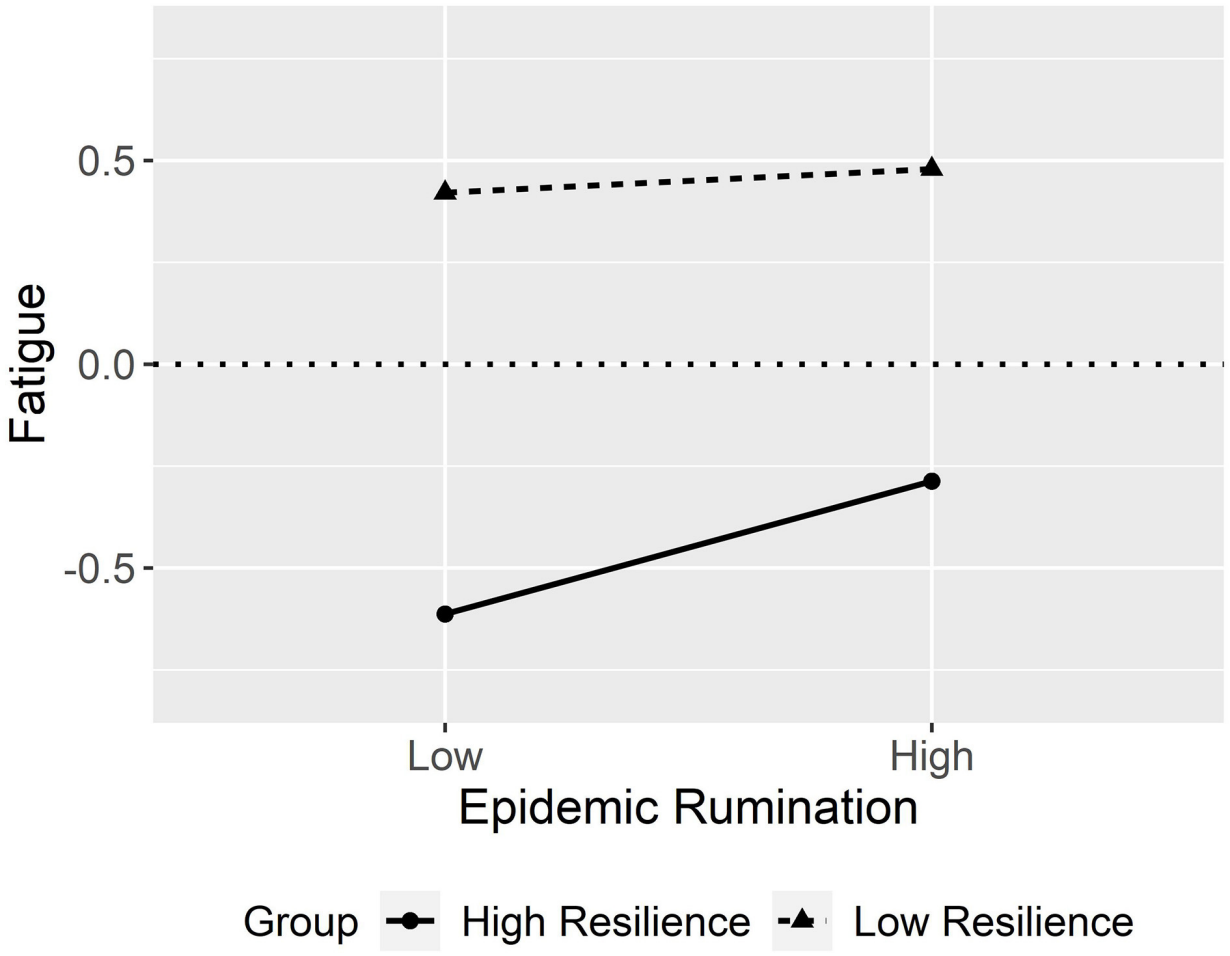
Interaction plot between epidemic rumination and resilience on fatigue.

The interaction effect is visually outlined in Figure 3 as a simple slopes plot with calculated gamma coefficients at −1 *SD* and + 1 *SD* from the mean of resilience. For students with low resilience, the impact of epidemic rumination on fatigue was not significant (γ = 0.03, *t* = 1.18, *p* > 0.05) compared to students with high resilience, where the impact of epidemic rumination on fatigue was significant (γ = 0.18, *t* = 5.54, *p* < 0.001). While this interaction effect was significant, the direction of the contrasted with the hypothesis, and thus Hypothesis 3 was rejected.

### Considering Alternative Models

Although results have generally provided strong support for our current model, several possible alternative models were also considered and tested given the cyclical nature of mental health outcomes and maladaptive behaviors (Table 2). Alternative Models I-II were direct derivatives of the proposed conceptual model but made strong assumptions that depressive symptoms were not directly preceded by resilience (Model I) and epidemic rumination (Model II). Both alternative models yielded comparable fits but ultimately did not allow for incorporating past findings that implicate direct effects of rumination [e.g., (15, 25)] and resilience on depressive symptoms [e.g., (30, 32, 35, 36)]. Further, consistent with prior evidence of resilience as a moderating trait [e.g., (26, 67)], Alternative Models IV-VIII were also examined in which rumination was tested as a moderator at multiple paths. However, none of these competing models yielded comparatively good or better fits.

**Table 2:** Comparison of alternative models.

**Model**	**Diagram**	**RMSEA**	**CFI**	**NNFI**	**SRMR**
Proposed model	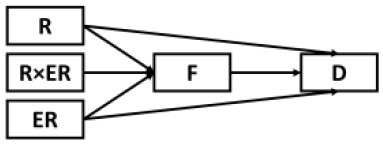	0.05	0.98	0.97	0.04
Alternative model I	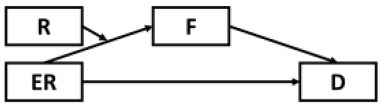	0.05	0.97	0.96	0.05
Alternative model II	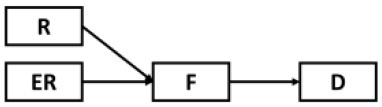	0.06	0.98	0.97	0.05
Alternative model III	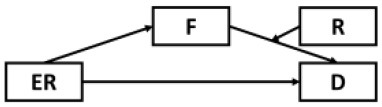	0.10	0.91	0.98	0.12
Alternative model IV	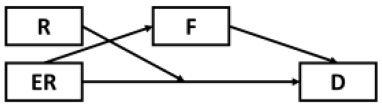	0.10	0.91	0.88	0.11
Alternative model V	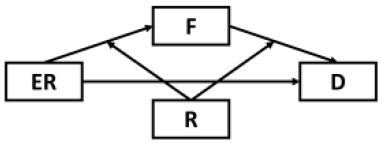	0.09	0.90	0.88	0.08
Alternative model VI	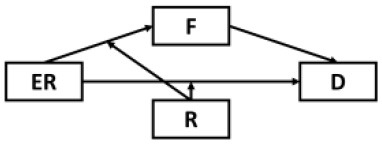	0.09	0.91	0.89	0.07
Alternative model VII	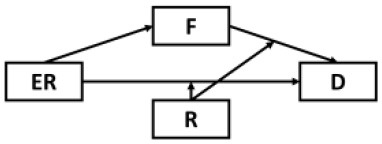	0.09	0.89	0.87	0.11
Alternative model VIII	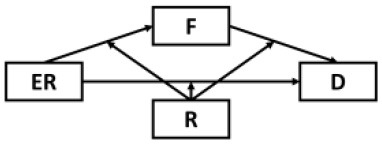	0.08	0.90	0.88	0.08
Alternative model IX	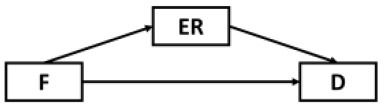	0.13	0.90	0.87	0.09
Alternative model X	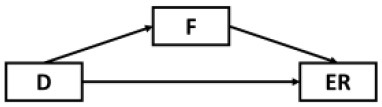	0.13	0.91	0.88	0.09
Alternative model XI	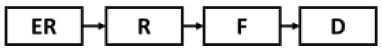	0.10	0.92	0.90	0.09
Alternative model XII	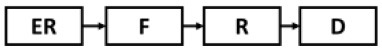	0.10	0.93	0.90	0.09

*ER, epidemic rumination; F, fatigue; D, depressive symptoms; R, resilience*.

Lastly, four models that restructured the order of variables were considered. Although epidemic rumination may be a risk factor for fatigue and depressive symptoms, prior evidence suggests that possibility of the opposite trend. Specifically, fatigue may hinder self-control (47), possibly leading to greater rumination and subsequently depressive symptoms [(51, 68) Alternative Model IX]. Similarly, depressive symptoms and rumination may also be cyclical in which depressive symptoms may induce greater focusing on negative emotions (24) that lead to both fatigue and rumination (Alternative Model X). Further, while resiliency is often depicted as a stable individual trait, recent findings have alluded that one's resiliency may be malleable in response to varying degrees of risk [e.g., (69)], as also possibly evidenced by the negative correlations of epidemic rumination and fatigue on resilience. Thus, Alternative Models XI-XII were examined to test whether epidemic rumination posed a direct effect on resilience or indirect effect via fatigue. However, all competing models yielded poorer fits in comparison to the proposed model. Hence, the proposed model best yielded empirical support for the conceptual path model.

## Discussion

Sudden public health emergencies risk serious social harm to the affected populace (70), particularly for college students who may be ill-equipped to adaptively manage the sudden stress of emergencies (71–73). This current research explored the effects of epidemic rumination and resilience on college students' depressive symptoms, the interaction between rumination and resilience, as well as the mediating role of fatigue. In this study, epidemic rumination was positively related to depressive symptoms, in line with several studies also documenting a positive link between the two constructs [e.g., (4, 17, 18, 58, 74)]. Similarly, resilience was negatively related to depressive symptoms, consistent with prior studies [e.g., (30, 31, 75)]. However, in examining the direct effects, fatigue was the strongest predictor of depressive symptoms, eclipsing the effect sizes of the two aforementioned predictors in comparison. This is not entirely unexpected, given that psychomotor retardation has long been a known sign of depression (76) and psychological and physiological exhaustion are close sister constructs. From the perspectives of the psychological resources theory (47) and cognitive load theory (77), fatigued individuals, depleted of psychological resources, may struggle in their fight against the onset of stress consequences when faced with prolonged negative emotional or psychological states, increasing the risk of depression (48, 49, 58, 78).

It is worth noting that in this study, the measurements used were meant to conceptually capture different components of a similar construct; fatigue measured one's extent of mental and psychological tiredness and exhaustion compared to psychomotor retardation that captures the physiological symptomology of depleted motivation in clinical depression. However, the two constructs remain fairly similar in their conceptualization and future research may seek to further parse them apart by specifying the target of the fatigue (i.e., fatigue over COVID-19 and its related events). For instance, the psychological and physiological consequences of prolonged exposure to COVID-19 information is popularly being referred to as the “COVID fatigue.” It is currently unclear whether target-specific fatigue may yield different results. However, should there be strong theoretical or empirical reasoning to suggest that “COVID fatigue” may result in greater consequences, such as evidence of avoidant coping specific to COVID-19 stressors, then future research may be warranted to examine this link.

In predicting fatigue, both resilience and epidemic rumination were significant correlates. Resilience was a strong negative correlate of fatigue whereas epidemic rumination was a small positive correlate of fatigue. In both cases, the results generally supported prior findings [e.g., (79)]. What was interesting, and somewhat counterintuitive, however, was the positive interaction between epidemic rumination and resilience. This result was in direct contradiction to our hypothesized direction that high resiliency would be buffer the effect of rumination on fatigue. One explanation may be that for those with very high levels of rumination, the negative effects were beyond the capacity of their ability to adequately cope. Indeed, while the conventional view has been that resilience serves as a protective role against difficulties, traumas, and tragedies (26, 67, 80), there has been a notable contention of scholars who have challenged this view, arguing that the benefits of resilience wanes at the highest levels of risk (69, 81, 82). For instance, Vanderbilt-Adriance and Shaw (69) suggested that the efficacy of protective factors can be lost when the counteracting risk surpasses a certain threshold. Thus, individuals who are highly resilient, but also ruminative, may continue to expend cognitive resources in spite of their inability to manage their stressors, exacerbating what may be an inevitable state of exhaustion. This may be in comparison to less resilient but ruminative individuals who may prefer the path of least resistance and simply let rumination work its course on inducing “normative” fatigue.

Although the interaction effect was notably small, fairly inconspicuous small effects may still yield long-term practical significant ramifications (83). Thus, future development and implementation of any interventions in building resilience may need to be more cognizant about possible unintended consequences toward those under high risk. Lastly, fatigue partially mediated the effects of epidemic rumination and resilience on depressive symptoms. This is significant in that mitigating physiological and psychological exhaustion may improve mental health outcomes. However, given that both epidemic rumination and resilience still yielded significant direct effects on depressive symptoms, targeted interventions may need to address several factors to observe large improvements in one's mental health outcomes.

### Significance and Implications of Research

While the current study did not directly examine the efficacy of any intervention strategies, the results provide several implications for what future studies may need to address. Firstly, it may be beneficial for college students to learn specific coping strategies. Given that COVID-19 is largely outside one's immediate control, certain active coping strategies that seek to address the source of the problem may not be practical or feasible. We also hesitate in advocating for any coping strategies that involve diverting one's attention from COVID-19 related contents as such endeavors may only serve to teach and promote avoidant coping. Thus, future research may seek to examine if strategies that address one's subjective response to stressors (e.g., cognitive reappraisal) prove fruitful in mitigating the onset of fatigue and depressive symptoms. Secondly, interventions that help college students to ensure adequate sleep, improve sleep quality, and engage in non-exhaustive exercise may hold potential benefits by reducing fatigue. Lastly, based on our findings that high resilience may not always yield desired outcomes, it may be necessary for interventions to first target reducing rumination prior to attempting to boost one's resilience.

### Limitations

Several limitations should be considered in interpreting the results. First, the cross-sectional and correlation study designs limit the extent to which causal inferences may be made. While alternative models were examined to compare contrasting theoretical paths and further justify the model examined, future studies should nonetheless seek to utilize longitudinal or experimental designs, as allowed, to further probe at the causality of the paths examined in this study. Secondly, the participants in the sample used were entirely from Chinese populations and may not generalize beyond this social ecology. Given that each country, and even the local clusters within geographical locations, may experience the COVID-19 pandemic differently, it is necessary for additional research to be conducted across cultures to examine the robustness of the model. Thirdly, all measures were examined via self-report scales. While statistical and process controls against common-methods bias were used (see Appendix for further details), future research may opt to incorporate mixed methods designs (e.g., quantitative with qualitative data, psychological with physiological measurements) to further enrich the findings from this study.

Fourthly, depressive symptomology may not necessarily translate to clinical depression. As was shown in Table 1, the mean score of depressive symptoms was far below the midpoint of the scale and most participants did not report the highest levels of symptoms to typically constitute clinical depression. Thus, while the analyses used in this study examine *relations* between variables, and thus are not affected by the location of the means, future research may seek to pursue replication studies on clinically diagnosed samples. Lastly, the current study only examined fatigue as a mediating variable. As evidenced by prior studies, several other mediating variables may be relevant as well. Future studies may seek to examine a more comprehensive model in explaining the antecedents of depressive symptoms.

## Conclusion

The current study provides novel insight into examining the roles of epidemic rumination, resilience, and fatigue on depressive symptoms. It is imperative to continue monitoring the well-being of college students as they reach key developmental milestones amidst an uncertain social ecology. While focusing intervention strategies on fatigue may yield the largest, direct benefit, attention should also be given to mitigating ruminative tendencies as well as promoting resiliency. This may particularly be important given the current finding that only promoting one factor in the absence of the other may result in exacerbating fatigue for select individuals.

## Data Availability Statement

The raw data supporting the conclusions of this article will be made available by the authors, without undue reservation.

## Ethics Statement

The studies involving human participants were reviewed and approved by Jiangxi Normal University. The patients/participants provided their written informed consent to participate in this study.

## Author Contributions

BY acted as the Principal Investigator and oversaw the study in its inception to completion. BY, XZ, ML, XW, and QY were responsible for data collection, writing the manuscript, and conceptualizing the models. HI contributed to the reconceptualization of the study models and rewriting of the paper in subsequent drafts after the initial submission. All authors contributed to the article and approved the submitted version.

## Conflict of Interest

The authors declare that the research was conducted in the absence of any commercial or financial relationships that could be construed as a potential conflict of interest.

## Appendix

### Addressing Common-Methods Bias

To control for common-method bias stemming from self-report measures, several steps were undertaken. To provide procedural control, participants' responses were anonymous and were free to withdraw from the study at any time. Further, it was stressed that there were no “right” or “wrong” answers to the measures. To provide statistical control, Harman's single-factor test was used. Exploratory factor analysis was run for all items of variables with rotated principal component and 13 factors were extracted with eigenvalues <1. The first factor accounted for 38.58%, suggesting no significant common-method bias in the data (84).

### The Adapted COVID-19 Ruminative Response Scale

1. I often think about what causes COVID-19.
2. I often analyze the outbreak of COVID-19 and try to understand why I am depressed.
3. I often think about why COVID-19 turned out the way it did.
4. I often go away by myself and think about why I feel this way.
5. I often write down what I am thinking about and analyze it.
6. I often think about the COVID-19 epidemic, wishing it will get better.
7. I often wonder why I have these problems that others don't.
8. I often wonder why I can't handle things better.
9. I often analyze my personality to try to understand why I am depressed.
10. I often go someplace alone to think about my feelings.

^*^*Note*: English translations are given but has not been empirically tested in its English form.
